# Non-Markovian exceptional points by interpolating quantum channels

**DOI:** 10.1038/s41534-026-01205-2

**Published:** 2026-03-02

**Authors:** Wai Chun Wong, Bei Zeng, Jensen Li

**Affiliations:** 1 Centre for Metamaterial Research and Innovation, Department of Physics and Astronomy, University of Exeter, Exeter, EX4 4QL UK; 2 Department of Physics, The University of Texas at Dallas, Richardson, TX 75080 USA

**Keywords:** Optics and photonics, Physics

## Abstract

Exceptional points (EPs) occur in non-Hermitian systems where eigenvalues and eigenvectors coalesce. In open quantum systems, EPs are typically studied via non-Hermitian effective Hamiltonians or Liouvillians, but these omit the full generality of quantum dynamics, which requires description using quantum channels. Here, we introduce a general strategy for generating quantum-channel EPs in the single-qubit setting. We show that quantum channels naturally fall into two distinct phases, purely real or complex-conjugate eigenvalues, without the need of enforcing additional symmetries, and interpolating between different phases induces a phase transition where EPs can emerge. We experimentally simulate such channels on a nuclear magnetic resonance quantum computer, observing second-order quantum-channel EPs with 93% fidelity, and extend the approach to three channels to uncover exceptional lines and a third-order quantum-channel EP. Our results promise potential applications from enhanced quantum sensing to quantum control, such as Jordan-chain mediated asymmetric conversion.

## Introduction

Non-Hermitian physics, which describes systems with dissipation and amplification, has gained significant attention in recent years^1–5^. A key example is parity-time (${\mathcal{P}}{\mathcal{T}}$)-symmetric systems, where balanced gain and loss enable real eigenvalues despite non-Hermiticity, leading to exceptional points (EPs) where eigenvalues and eigenvectors coalesce^6,7^. EPs have been widely studied in optics, acoustics, and electronic systems, enabling unidirectional invisibility, novel lasing behaviors, and enhanced sensing^8–20^. In passive systems, introducing a global loss bias allows PT-like behavior without requiring gain^8^, while state evolution around EPs exhibits path-dependent transport effects^21,22^.

Recently, EPs have been extended to open quantum systems, with demonstrations of chiral state transfer in trapped ions and cold atoms^23–25^. These implementations often rely on engineering an effective PT-symmetric Hamiltonian to obtain Hamiltonian exceptional points (HEPs), but this approach does not fully capture quantum dynamics in the presence of decoherence or noise^26–28^. A more complete description requires the Lindblad master equation, where EPs emerge in the non-Hermitian Liouvillian superoperator, termed Liouvillian exceptional points (LEPs). Unlike HEPs, LEPs can arise even when no corresponding HEPs exist, offering new opportunities for quantum state control^29–32^. Experimental realizations of LEPs have been reported in trapped ions, superconducting qubits, and quantum computers^27–35^. However, these studies generally assume Markovian environments to arrive at a well-defined Lindblad master equation, whereas recent work has proposed EPs in non-Markovian regimes by considering a structured reservoir with memory effect, where the Liouvillian deviates from the traditional Lindblad form^36^.

Here, we propose interpolating quantum channels as a versatile framework for establishing EPs in open quantum systems^37^. These quantum channels are completely positive and trace-preserving (CPTP) in a single step but are generally not CP-divisible, indicating another way to have non-Markovian dynamics. Consequently, their dynamics do not obey the time-independent Lindblad master equation with CP-divisible Liouvillian that most work on LEP is based on.

Notably, such a quantum channel is guaranteed to exist in one of two distinct phases—one characterized by purely real eigenvalues and the other by complex conjugate pairs—without requiring symmetry constraints such as PT symmetry. By interpolating between quantum channels from different phases, EPs naturally emerge at the transition, providing a systematic method for generating them. We apply this approach to a single-qubit channel to construct both second- and third-order EPs, corresponding to the interpolation of two and three channels, respectively. In the latter case, EP lines also emerge. Using quantum process tomography (QPT), previously employed to identify LEPs^35^, we demonstrate non-Markovian EPs realized through our channel interpolation approach on a nuclear magnetic resonance (NMR) quantum computer.

## Results

Open quantum systems interact with their environment, leading to decoherence and information loss beyond unitary dynamics. The Markovian approximation captures these effects through the Lindblad master equation^38^ $d\rho /{dt}={\mathcal{L}}\rho$, where the Liouvillian superoperator ${\mathcal{L}}$ governs how quantum states evolve under environmental influence, as a CPTP generator. For a single qubit, this evolution can be represented by a 4 × 4 non-Hermitian matrix acting on vectorized density matrix $\{{\rho }_{11},{\rho }_{21},{\rho }_{12},{\rho }_{22}\}$. This non-Hermitian structure allows for the emergence of LEPs: parameter values where eigenvalues and eigenvectors coalesce, fundamentally altering the system’s dynamics. In this work, we adopt the definition from ref. ^39^. that treats dynamics as Markovian when generated by a time-independent CPTP generator ${\mathcal{L}}$, meaning the Liouvillian superoperator remains constant over time. This definition aligns with the literature on LEPs, which predominantly considers them in this time-independent Markovian framework, while we extend this exceptional point physics beyond Markovian dynamics to general quantum channels.

While Lindbladian evolution assumes memoryless (Markovian) environments, quantum channels ${\mathcal{E}}$ describe a broader range of quantum state transformations, including those with memory effects, non-Markovian noise, and measurement backaction. By definition, a quantum channel ${\mathcal{E}}$ is a map satisfying complete positivity and trace preservation (CPTP):

1 $$\left(\begin{array}{l}{\rho }_{11}^{{\prime} }\\ {\rho }_{21}^{{\prime} }\\ {\rho }_{12}^{{\prime} }\\ {\rho }_{22}^{{\prime} }\end{array}\right)={\mathcal{E}}\left(\begin{array}{l}{\rho }_{11}\\ {\rho }_{21}\\ {\rho }_{12}\\ {\rho }_{22}\end{array}\right),$$

with the transformed density matrix $${\rho }^{{\prime} }=\left(\begin{array}{cc}{\rho }_{11}^{{\prime} } & {\rho }_{12}^{{\prime} }\\ {\rho }_{21}^{{\prime} } & {\rho }_{22}^{{\prime} }\end{array}\right)$$. Here, we assumed the mapping is linear, such that the map can be represented by the above matrix multiplication in terms of linear algebra. We note that this is not the standard Choi representation, in which the CPTP condition would be transparent. The benefit of this natural representation (or superoperator representation) is its close relationship to LEPs in ref. ^29^. as follows. For Markovian evolution, ${\rho }^{{\prime} }$ represents the transformed density matrix after a fixed time *T*, and the channel can then be written in terms of a time-independent ${\mathcal{L}}$ with the form ${\mathcal{E}}={e}^{{\mathcal{L}}T}$. This connection motivates us to introduce channel exceptional points as degeneracies in the eigenvalues and eigenvectors of the channel map ${\mathcal{E}}$ itself. This generalization is natural because every LEP automatically induces a channel exceptional point through such an exponential map. However, many quantum channels cannot be generated by a CPTP (Lindblad) generator. Such channels fall outside the class of CP-divisible dynamics and are commonly regarded as non-Markovian. Nevertheless, they can still exhibit exceptional points. This is because exceptional points are determined by the spectral properties of the linear dynamical map itself, which remains well defined even if the Liouvillian is not well defined (e.g. not CP-divisible). This extension opens new territory. While LEPs are confined to Markovian dynamics, channel exceptional points exist across all CPTP maps, including non-Markovian processes and measurement-induced evolution. By studying exceptional points at the channel level, we gain access to a broader landscape of quantum degeneracies.

To reveal two distinct phases of a quantum channel, we change the basis^40^ in Eq. (1) to

2 $$\left(\begin{array}{c}1\\ {r}_{x}^{{\prime} }\\ {r}_{y}^{{\prime} }\\ {r}_{z}^{{\prime} }\end{array}\right)=\left(\begin{array}{cccc}1 & 0 & 0 & 0\\ {s}_{x} & {E}_{{xx}} & {E}_{{xy}} & {E}_{{xz}}\\ {s}_{y} & {E}_{{yx}} & {E}_{{yy}} & {E}_{{yz}}\\ {s}_{z} & {E}_{{zx}} & {E}_{{zy}} & {E}_{{zz}}\end{array}\right)\,\left(\begin{array}{c}1\\ {r}_{x}\\ {r}_{y}\\ {r}_{z}\end{array}\right)$$

Here, the quantum channel is represented by transforming Bloch vector components ${r}_{x}={\rho }_{12}+{\rho }_{21}$, ${r}_{y}=i\left({\rho }_{12}-{\rho }_{21}\right)$ and ${r}_{z}={\rho }_{11}-{\rho }_{22}$. Since these components, along with the first component ${\rm{Tr}}\left(\rho \right)=1={\rho }_{11}+{\rho }_{22}$ are linear in terms of |*ρ*»$=${*ρ*_11_, *ρ*_21_, *ρ*_12_, *ρ*_22_}, the transformation matrix is given by $M{\mathcal{E}}{M}^{-1}$ with an invertible matrix, and they share the same eigenvalues and channel EPs. For simplicity, we will also refer to this matrix as ${\mathcal{E}}$ when the context is clear. The first row, fixed as (1,0,0,0) due to the trace-preserving condition, ensures a trivial eigenvalue of 1 and other eigenvalues given by eigenvalues of submatrix *E*. Geometrically, this distortion matrix *E* maps the Bloch vector of state *ρ* and is shifted by the shift vector *s*. Since ${\mathcal{E}}$ is now a real matrix, its eigenvalues appear in conjugate pairs, as ${\mathcal{E}}v=\lambda v$ implies ${{\mathcal{E}}}^{* }{v}^{* }={\mathcal{E}}{v}^{* }={\lambda }^{* }{v}^{* }$ where * represents complex conjugate. The eigenvalues can be either fully real, $\left\{1,\,{\lambda }_{1},\,{\lambda }_{2},{\lambda }_{3}\right\}$ $\in$ ${\mathbb{R}}$ or form one conjugate pair i.e., $\{1,{\lambda }_{1}{\mathbb{\}}}{\mathbb{\in }}{\mathbb{R}}$, $\left\{{\lambda }_{2},{\lambda }_{2}^{* }\right\}{\mathbb{\in }}{\mathbb{C}}$.

It is easy to show that in the first case, all eigenvectors are real, while in the second, the eigenvector of *λ*_1_ is real, and those of $\left\{{\lambda }_{2},{\lambda }_{2}^{* }\right\}$ form a conjugate pair. This mirrors the behavior of ${\mathcal{P}}{\mathcal{T}}$-symmetric systems, where eigenvalues are real in the exact phase and complex in the broken phase. However, in our case, these phases arise from the real nature of ${\mathcal{E}}$ (${\mathcal{E}}={{\mathcal{E}}}^{* }$) rather than an additional requirement of an engineered gain-loss balance in ${\mathcal{P}}{\mathcal{T}}$-symmetric systems. To distinguish this from ${\mathcal{P}}{\mathcal{T}}$-symmetry, we introduce the complex conjugation operator ${\mathcal{K}}$, which emphasizes the real nature of the channel rather than physical time reversal. We have ${\mathcal{K}}E{{\mathcal{K}}}^{-1}=E$. This allows us to classify the system’s phases as the ${\mathcal{K}}$-exact phase, where eigenvalues and eigenvectors are real, and the ${\mathcal{K}}$-broken phase, where eigenvalues and eigenvectors form complex conjugate pairs. For completeness, one can also construct a real symmetric matrix *η* satisfying pseudo-Hermiticity $E={\eta }^{-1}{E}^{\dagger }\eta$ to have the same eigenvalue structures. However, such *η* is generally not a fixed matrix with different *p*.

### EP from interpolating two quantum channels

**Fig. 1:**
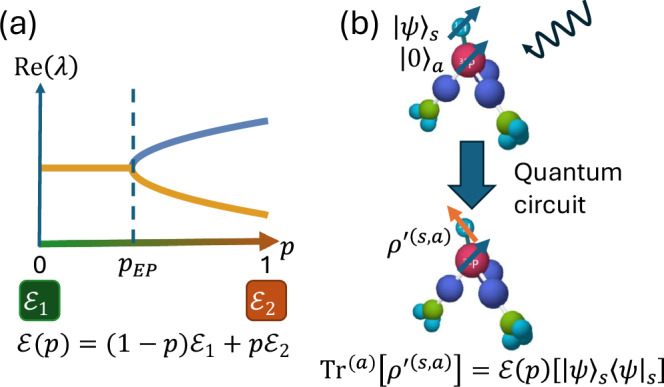
Exceptional points in interpolated quantum channels via nuclear magnetic resonance (NMR). **a** Schematic of linear interpolation between two quantum channels to generate a family of quantum channels with exceptional points. Not all eigenvalues are included for clarity. Two quantum channels ${{\mathcal{E}}}_{1}$ and ${{\mathcal{E}}}_{2}$ are connected by a linear interpolation ${\mathcal{E}}\left(p\right)=\left(1-p\right){{\mathcal{E}}}_{1}+p{{\mathcal{E}}}_{2}$ with *p*$\in$[0, 1]. The exceptional point is located at *p*=*p*_*EP*_ where the two eigenvalues of the superoperator ${\mathcal{E}}(p)$ coalesce. **b** Simulation of the quantum channels with a circuit-based NMR quantum computer. The molecule Dimethylphosphite ((CH_3_O)_2_PH) provides ^31^P (red) and ^1^H (cyan) nuclei as the two qubits in the NMR quantum computer. Two qubits are separated as signal and ancilla qubits. The signal qubit and ancilla qubit are initialized to the state |*ψ*〉_*s*_ and |0〉_*α*_, respectively. After the quantum circuit, the signal qubit is measured to obtain the output state of the quantum channel ${\mathcal{E}}\left(p\right){|\psi \rangle }_{s}{\langle \psi |}_{s}$.

To examine the phase transition, we consider a linear interpolation between two quantum channels, ${{\mathcal{E}}}_{1}$ and ${{\mathcal{E}}}_{2}$ with different phases, as shown in Fig. 1a:

3 $${\mathcal{E}}\left(p\right)=\left(1-p\right){{\mathcal{E}}}_{1}+p{{\mathcal{E}}}_{2},$$

where $p\,\in \,\left[0,1\right]$. Physically, this represents a state entering ${{\mathcal{E}}}_{1}$ with probability 1 − *p* and ${{\mathcal{E}}}_{2}$ with probability *p*. As an example, we consider two quantum channels with vanishing shift vector *s* = 0 (unital), where their distortion matrix *E* is given by

4 $${E}_{1}=\frac{1}{2}\left(\begin{array}{ccc}0 & 0 & 0\\ 0 & 0 & -1\\ 0 & 1 & 0\end{array}\right),\,{E}_{2}=\frac{1}{2}\left(\begin{array}{ccc}0 & 0 & 0\\ 0 & 1 & 0\\ 0 & 0 & -1\end{array}\right)$$

These channels are chosen for their simplicity and their distinct phases (the first channel has complex conjugate eigenvalues). While their complete positivity (CP) is not immediately apparent from the matrix form, it becomes clear from their Kraus representations:

5 $$\begin{array}{l}{{\mathcal{E}}}_{1}\left[\rho \right]=\frac{1}{2}\sqrt{{\sigma }_{x}}\rho {\sqrt{{\sigma }_{x}}}^{\dagger }+\frac{1}{4}{\sigma }_{y}\rho {\sigma }_{y}+\frac{1}{4}{\sigma }_{z}\rho {\sigma }_{z},\\ {{\mathcal{E}}}_{2}\left[\rho \right]=\frac{1}{4}\rho +\frac{1}{4}{\sigma }_{x}\rho {\sigma }_{x}+\frac{1}{2}{\sigma }_{y}\rho {\sigma }_{y}.\end{array}$$

Since each term is CP, their sum is CP as well (TP can be seen from Eq. 2). To analyze the EPs in the interpolated quantum channel, we examine how the distortion matrix varies with *p*. By linearity, the distortion matrix of ${\mathcal{E}}\left(p\right)$ is

6 $$E\left(p\right)=\left(1-p\right){E}_{1}+p{E}_{2},=\frac{1}{2}\left(\begin{array}{rcl}0 & 0 & 0\\ 0 & p & p-1\\ 0 & 1-p & -p\end{array}\right).$$

The eigenvalues are *λ*_1_ = 0 and ${\lambda }_{\pm }=\pm \sqrt{\frac{p}{2}-\frac{1}{4}}$, with eigenvectors ${{\boldsymbol{r}}}_{1}=\left\{1,0,0\right\}$, ${{\boldsymbol{r}}}_{\pm }=\{0,p+2{{\rm{\lambda }}}_{\pm },1-p\}$. Both eigenvectors coalesce at *p* = 1/2, to ${{\boldsymbol{r}}}_{{\rm{EP}}}=\left\{\mathrm{0,1,1}\right\}/\sqrt{2}$ (normalized), indicating an exceptional point. In this case, the quantum channel admits a Jordan chain: $E{{\boldsymbol{r}}}_{J}={\lambda }_{{EP}}{{\boldsymbol{r}}}_{J}+{{\boldsymbol{r}}}_{{EP}}/2,\,E{{\boldsymbol{r}}}_{{EP}}={\lambda }_{{EP}}{{\boldsymbol{r}}}_{{EP}},$ with *λ*_*EP*_ = 0. Choosing the Jordan chain generator ${{\boldsymbol{r}}}_{J}=(0,1,-1)/\sqrt{2}$, which is orthogonal to the coalesced eigenvector ***r***_*EP*_, a non-zero input ***r***_*J*_ is mapped to a non-zero output along ***r***_*EP*_ while an input aligned with ***r***_*EP*_ is mapped to zero, establishing a strictly one-way asymmetry between the two directions of the Jordan chain. This asymmetric conversion behavior, arising from laddering along a Jordan chain, can in principle be verified by preparing the associated density matrices in an open quantum system and shares the same physical origin as chiral mode excitation and emission at EPs in optical and acoustic systems^41,42^.

The non-Markovian property of this channel EP is rather subtle. The usual method of showing all possible ${\mathcal{L}}$ such that ${\mathcal{E}}\left(1/2\right)={e}^{{\mathcal{L}}}$ yield invalid Lindblad master equation cannot apply here due to the zero eigenvalues of ${\mathcal{E}}\left(1/2\right)$. Instead, we show that ${\mathcal{E}}\left(1/2\right)$ can be approximated arbitrarily closely by non-Markovian channels. We start by stating that the distortion matrix at this EP is given by

7 $${E}_{{\rm{EP}}}=E\left(1/2\right)=\frac{1}{4}\left(\begin{array}{ccc}0 & 0 & 0\\ 0 & 1 & -1\\ 0 & 1 & -1\end{array}\right),$$

with its Jordan normal form:

8 $${E}_{{\rm{EP}}}=\left(\begin{array}{ccc}1 & 0 & 0\\ 0 & 1 & 2\\ 0 & 1 & -2\end{array}\right)\left(\begin{array}{ccc}0 & 0 & 0\\ 0 & 0 & 1\\ 0 & 0 & 0\end{array}\right){\left(\begin{array}{ccc}1 & 0 & 0\\ 0 & 1 & 2\\ 0 & 1 & -2\end{array}\right)}^{-1},$$

i.e., *E*_EP_ is similar to the direct sum of a 1 × 1 Jordan block with eigenvalue 0 and a 2 × 2 Jordan block with eigenvalue 0. To obtain an invertible quantum channel, we approximate the channel while preserving the Jordan structure by replacing the zero eigenvalues with *e*^−*γ*^:

9 $${E}_{{\rm{EP}}}\left(\gamma \right)=\left(\begin{array}{ccc}1 & 0 & 0\\ 0 & 1 & 2\\ 0 & 1 & -2\end{array}\right)\left(\begin{array}{ccc}{e}^{-\gamma } & 0 & 0\\ 0 & {e}^{-\gamma } & 1-{e}^{-\gamma }\\ 0 & 0 & {e}^{-\gamma }\end{array}\right){\left(\begin{array}{ccc}1 & 0 & 0\\ 0 & 1 & 2\\ 0 & 1 & -2\end{array}\right)}^{-1}$$

The original channel is recovered in the limit *γ* → ∞, where the zero eigenvalues emerge from exponential decay. The term 1−*e*^−*γ*^ in the (2,3) matrix element ensures the channel remains valid for all γ as it is the sum (1−e^−γ^)E_*EP*_+e^−γ^I. For a finite *γ*, the only candidate Liouvillian superoperator is $\mathrm{ln}{E}_{{\rm{EP}}}\left(\gamma \right)$ in the principal branch (other branches yield complex Liouvillian superoperator, violating Hermiticity preservation in the resulting Lindblad master equation):

10 $${\mathrm{ln}}\,{E}_{\mathrm{EP}}\left(\gamma \right)=\left(\begin{array}{rcl}1 & 0 & 0\\ 0 & 1 & 2\\ 0 & 1 & -2\end{array}\right)\left(\begin{array}{rcl}-\gamma & 0 & 0\\ 0 & -\gamma & {{\rm{e}}}^{\gamma }\left(1-{e}^{-\gamma }\right)\\ 0 & 0 & -\gamma \end{array}\right){\left(\begin{array}{rcl}1 & 0 & 0\\ 0 & 1 & 2\\ 0 & 1 & -2\end{array}\right)}^{-1}$$

However, this Liouvillian becomes invalid for large *γ*. To show this, we solve the Lindblad master equation:

11 $${e}^{t\,{\mathrm{ln}}\,{E}_{\mathrm{EP}}\left(\gamma \right)}=\frac{1}{4}\left(\begin{array}{rcl}4{e}^{-t\gamma } & 0 & 0\\ 0 & \left(4-t\right){e}^{-t\gamma }+t{e}^{\left(1-t\right)\gamma } & t{e}^{-t\gamma }-t{e}^{\left(1-t\right)\gamma }\\ 0 & -t{e}^{-t\gamma }+t{e}^{\left(1-t\right)\gamma } & \left(4+t\right){e}^{-t\gamma }-t{e}^{\left(1-t\right)\gamma }\end{array}\right)$$

For the intermediate evolution time *t* ∈ (0,1), several matrix elements grow without bound as *γ* increases. When *γ* becomes sufficiently large, this “quantum channel” expands the Bloch sphere and fails to preserve positivity of input states. Therefore, the EP channel ${\mathcal{E}}\left(1/2\right)$ is a limit of non-Markovian EPs. In fact, the quantum channel ${\mathcal{E}}\left(p\right)$ for *p* around the EP is non-Markovian.

Although we choose the quantum channels ${{\mathcal{E}}}_{1}$ and ${{\mathcal{E}}}_{2}$ for simplicity, the concept extends generally. Linear interpolation between any two quantum channels with different phases inevitably leads to phase transitions where eigenvalues coalesce, indicating potential EPs. At these transitions, two cases arise: true EPs, where both eigenvalues and eigenvectors coalesce, and Diabolic Points (DPs), where only eigenvalues merge while eigenvectors remain distinct. This insight suggests a practical approach for generating EPs by systematically testing pairs of quantum channels with different phases. If a transition results in a DP rather than an EP, the same interpolation strategy can be applied to alternative channel pairs to access EPs. Given the vast number of quantum channels and the simplicity of this procedure, this provides an efficient method for generating EPs in quantum channels.

### Simulation of a quantum channel with a quantum computer

**Fig. 2:**
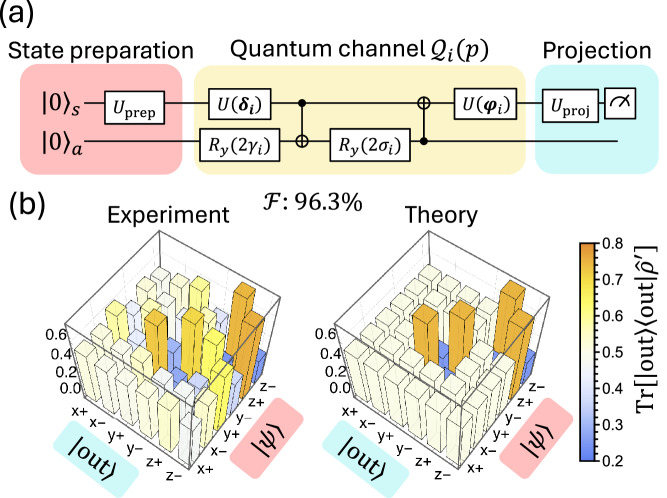
Circuit implementation and tomography of quantum channels. **a** Circuit for the implementation of the quantum channels ${\mathcal{E}}(p)$ and process tomography. Each quantum channel ${\mathcal{E}}(p)$ is implemented by splitting into two simpler quantum channels ${{\mathcal{Q}}}_{1}\left(p\right),\,{{\mathcal{Q}}}_{2}\left(p\right)$ which can be implemented using 2 qubits with U_3_ gates, R_y_ gates, and CNOT gates shown in the middle. The process tomography is performed by preparing the input state |*ψ*_*s*_〉 into eigenstates of Pauli operators, i.e., $\left\{\left|{x}_{\pm }\right\rangle ,\left|{y}_{\pm }\right\rangle ,\left|{z}_{\pm }\right\rangle \right\}$, using U_prep_ and measuring Pauli observables using U_proj_. **b** Experimental results of the measurement for the quantum channels ${\mathcal{E}}(p=1)$ and the theory prediction. We obtain a process fidelity of 96.3% for the experimental result.

To experimentally simulate the exceptional point, we use a two-qubit NMR circuit-based quantum computer^43^ (SpinQ Gemini, for 2-qubit quantum circuits) to implement the quantum channels ${\mathcal{E}}(p)$ and retrieve their eigenvalues. The standard approach for implementing a single-qubit quantum channel, Stinespring dilation^44^, requires two additional ancillary qubits (totally 3 needed) and a generic three-qubit unitary gate. Due to the high sensitivity of EPs to perturbations, together with the relatively deep circuits (on the order of tens of two-qubit entangling gates) resulting from the decomposition of a generic three-qubit unitary, the accumulated error and decoherence can be non-negligible^35^. While error-mitigation techniques and advances in hardware could alleviate these noise issues, we instead follow the method from ref. ^45^ decomposing ${\mathcal{E}}(p)$ into two simpler quantum channels ${{\mathcal{Q}}}_{1}(p)$, ${{\mathcal{Q}}}_{2}(p)$ for each *p* such that ${\mathcal{E}}\left(p\right)=1/2\left({{\mathcal{Q}}}_{1}\left(p\right)+{{\mathcal{Q}}}_{2}(p)\right)$ where each of these channels requires only one ancillary qubit (totally 2 qubits needed) and can be implemented using the quantum circuit shown in Fig. 2a. This circuit consists of two general unitary *U*_3_ gates ($U\left({\boldsymbol{\delta }}\right),U\left({\boldsymbol{\varphi }}\right)$) for diagonalizing the distortion matrix, along with two *R*_*y*_ and two CNOT gates to implement the shift vector and diagonalized distortion matrix. We implement ${{\mathcal{Q}}}_{1}(p)$ and ${{\mathcal{Q}}}_{2}(p)$ separately, averaging their measurement results to construct ${\mathcal{E}}(p)$. The algorithm for determining the circuit parameters is provided in the method section.

To obtain the eigenvalues of the quantum channel, we perform QPT to fully characterize the superoperator ${\mathcal{E}}(p)$. For each quantum channel ${\mathcal{E}}(p)$, we prepare six input states corresponding to the eigenstates of the Pauli operators ${\boldsymbol{\sigma }}=\left\{{\sigma }_{x},{\sigma }_{y},{\sigma }_{z}\right\}$, i.e., {|*x*_±_〉= (|0〉 ± |1〉)/$\sqrt{2}$, {|*y*_±_〉 = (|0〉 ± *i*|1〉)/ $\sqrt{2}$, |*z*_+_〉 = |0〉, |*z*_-_〉=|1〉}, achieved by applying appropriate state preparation unitary *U*_prep_. We then measure the output states in these same Pauli bases by applying a suitable measurement unitary *U*_proj_ before readout. The total circuit for the implementation of the quantum channels ${{\mathcal{Q}}}_{\mathrm{1,2}}\left(p\right)$ and the process tomography is shown in Fig. 2a.

**Fig. 3:**
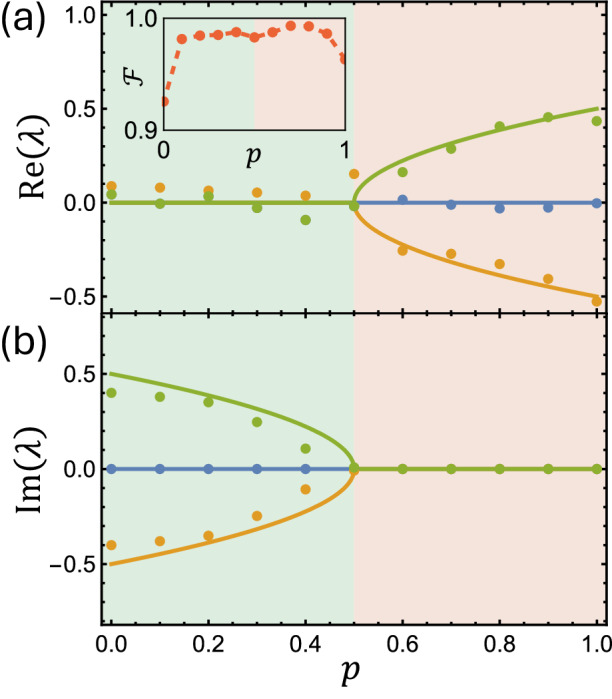
Eigenvalue spectra of the quantum channel. **a** The real and **b** imaginary parts of the eigenvalues of the superoperator ${\mathcal{E}}\left(p\right)$ as a function of *p*. The trivial eigenvalue 1 is omitted for clarity. The solid lines are the theoretical prediction, and the dots are the experimental results from the quantum process tomography. The exceptional point is located at *p* = 0.5 where the two eigenvalues coalesce. Inset: The fidelity of the experimental results with the theoretical prediction. The fidelity is above 93% for the whole range of *p*.

The experimental result for ${\mathcal{E}}\left(1\right)={{\mathcal{E}}}_{2}$ is shown in Fig. 2b with high agreement with the theory prediction. For the channel tomography, we use this result to perform maximum-likelihood fitting for a quantum channel with the CPTP condition^46^. We found that the process fidelity ${\mathcal{F}}$ of the experimental result with the theoretical prediction is 96.3%^47^. The fitting process and the definition of the fidelity of the quantum channel are also provided in the method section. After reconstructing the quantum channel ${\mathcal{E}}(p)$, we plot its eigenvalues as a function of *p* in Fig. 3. The experimental results are in good agreement with the theoretical prediction, and the exceptional point is located at $p=0.5$ where the two eigenvalues coalesce. The fidelity of the experimental results with the theoretical prediction is above 93% for the whole range of *p*, as shown in the inset of Fig. 3. This demonstrates the existence of exceptional points in the quantum channels ${\mathcal{E}}(p)$ and the successful implementation of the quantum channels using a circuit-based NMR quantum computer.

On top of process tomography shown here, EP dynamics may also be probed by revealing asymmetric conversion using quantum states. Importantly, the asymmetric conversion discussed here occurs at the level of Bloch rays—i.e., entire families of quantum states sharing the same Bloch direction—rather than merely between individual quantum states. At the EP ($p=1/2$) of $E(p)=(1-p){E}_{1}+p{E}_{2}$, the quantum channel ${\mathcal{E}}$ maps the Bloch ray associated with the Jordan vector ***r***_*J*_ onto the Bloch ray associated with the coalesced eigenvector ***r***_EP_, while the Bloch ray along ***r***_EP_ is mapped to the maximally mixed state, corresponding to a directionless fixed point in Bloch space. Consequently, the output state carries no Pauli component along the Jordan direction, i.e., ${Tr}\left({\mathcal{E}}\left(\rho \right){{\boldsymbol{r}}}_{J}\cdot {\boldsymbol{\sigma }}\right)=0$ for inputs on the Bloch ray along ***r***_EP_. This ray-level asymmetry can be probed experimentally by preparing representative states from each Bloch ray (e.g., pure states aligned with ***r***_*J*_ or ***r***_EP_) and measuring $\langle {\hat{r}}_{{\rm{EP}}}\,\cdot {\boldsymbol{\sigma }}\rangle$ and $\langle {\hat{r}}_{{\rm{J}}}\,\cdot {\boldsymbol{\sigma }}\rangle$ for the output state, revealing the intrinsically one-way, Jordan-chain–mediated conversion.

### Higher-order channel EP by interpolating three channels

The previous example focuses on interpolating 2 quantum channels. We can further extend our scheme to interpolate 3 quantum channels. With an extra channel, we now have one more interpolating parameter where we expect richer phenomena at the phase transition, such as higher-order exceptional point, as we shall see. We consider 3 quantum channels ${{\mathcal{E}}}_{1}$, ${{\mathcal{E}}}_{2}$ and ${{\mathcal{E}}}_{3}$ and interpolating them by

12 $${\mathcal{E}}\left({a}_{1},{a}_{2},{a}_{3}\right)={a}_{1}{{\mathcal{E}}}_{1}+{a}_{2}{{\mathcal{E}}}_{2}+{a}_{3}{{\mathcal{E}}}_{3}$$

with ${a}_{1}+{a}_{2}+{a}_{3}=1$. We choose the quantum channels ${{\mathcal{E}}}_{1}$ and ${{\mathcal{E}}}_{2}$ as same as before, and for the third unital quantum channel, we chose to have the distortion matrix ${{\mathcal{E}}}_{3}$ as a rotation matrix for the rotation along as an axis $\hat{n}=\{\mathrm{1,1,1}\}$ for an angle of −*π*/2 to further mix all three principal axes. By Rodrigues’ rotation formula, we have the distortion matrix ***E***_3_ as

13 $${E}_{3}=\frac{1}{3}\left(\begin{array}{ccc}1 & 1+\sqrt{3} & 1-\sqrt{3}\\ 1-\sqrt{3} & 1 & 1+\sqrt{3}\\ 1+\sqrt{3} & 1-\sqrt{3} & 1\end{array}\right)$$

**Fig. 4:**
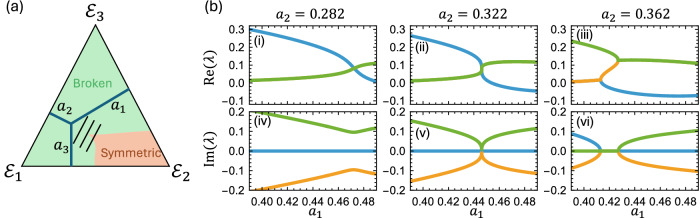
Exceptional point by interpolating 3 quantum channels. **a** Phase diagram for interpolating 3 quantum channels ${{\mathcal{E}}}_{1}$, ${{\mathcal{E}}}_{2}$ and ${{\mathcal{E}}}_{3}$ with distortion matrix *E*_1_, *E*_2_, and *E*_3_ in Eqs. (4) and (13). The interpolating parameters are *a*_1_, *a*_2_, and *a*_3_ under the constraint ${a}_{1}+{a}_{2}+{a}_{3}=1$ to ensure the resultant quantum channel ${\mathcal{E}}\left({a}_{1},{a}_{2},{a}_{3}\right)={a}_{1}{{\mathcal{E}}}_{1}+{a}_{2}{{\mathcal{E}}}_{2}+{a}_{3}{{\mathcal{E}}}_{3}$ is a valid quantum channel. The triangle represents the convex hull of these 3 quantum channels. There is an order 3 exceptional point EP_3_ located at $\left({a}_{1},\,{a}_{2},{a}_{3}\right)\cong (\mathrm{0.446,0.322,0.232})$. **b** The real (i), (iii), (v) and imaginary parts (ii), (iv), (vi) of the eigenvalues of the superoperator ${\mathcal{E}}\left({a}_{1},\,{a}_{2},{a}_{3}\right)$ as a function of *a*_1_ with ${a}_{2}=\{\mathrm{0.282,0.322,0.362}\}$ and ${a}_{3}=1-{a}_{1}-{a}_{2}$, i.e., three black lines in (**a**). These three values of *a*_2_ correspond to the three black lines shown in (**a**). The trivial eigenvalue 1 is omitted for clarity. At *a*_2 _= 0.282 (left black line in **a**), all points along this parametric path remain in the broken phase, with eigenvalues forming one conjugate pair and one real value. At *a*_2_ = 0.322 (middle line), the path passes through the convergence point, revealing the third-order exceptional point EP₃ in panels (ii, v). At *a*₂ = 0.362 (right line), the path crosses two exceptional lines, yielding two second-order exceptional points. This sequence demonstrates the coalescence of two EP₂ points into a single EP₃.

In Fig. 4a, we show the phase diagram of the 3 quantum channels interpolation, i.e., $E\left({a}_{1},{a}_{2},{a}_{3}\right)={a}_{1}{E}_{1}+{a}_{2}{E}_{2}+{a}_{3}{E}_{3}$. At the base of the equilateral triangle representing the parameter space (*a*_3_ = 0), we have the same linear interpolation as before, and the phase transition becomes now an exceptional line in the parameter space. We have calculated the phase rigidity and confirmed that these phase transition lines are indeed exceptional points, as at least one phase rigidity vanishes^48^. Moreover, we found that there is convergence of 2 phase transition lines at $\left({a}_{1}:{a}_{2}:{a}_{3}\right)=(10:2\sqrt{13}:3\sqrt{3})$. At this convergence point, we found the distortion matrix having 3 coalescing eigenvalues and eigenvectors. This is an order 3 exceptional point EP_3_. This EP_3_ channel is non-Markovian even under the general, time-dependent CP-divisibility definition, thus violating the weaker Markovian condition as well as the time-independent restriction adopted in this work. This is due to the smallest singular value *s*_min_ of the distortion matrix $E\left({a}_{1},{a}_{2},{a}_{3}\right)$ at the EP satisfying ${s}_{\min }^{2} < \det E$ a necessity and sufficiency condition given by ref. ^49^. Here, we do not perform the experiment for the 3 quantum channels interpolation, but the experimental implementation is feasible and can be done using the same method as the 2 quantum channels interpolation. In Fig. 4b, we show the real and imaginary parts of the eigenvalues of the quantum channel ${\mathcal{E}}({a}_{1},{a}_{2},{a}_{3})$ as a function of *a*_1_ and fixing *a*_2_ around the exceptional point. We can observe that the 2 second-order exceptional points coalesce and form a third-order exceptional point.

## Discussion

We should note that our work can be extended to higher-dimensional quantum channels. In fact, the Pauli basis used to describe the quantum channel has a natural extension to higher dimensions. For example, in the two-qubit case, the Pauli basis consists of 16 operators, including the identity operator and 15 tensor products of Pauli operators. The quantum channel can be represented as a 16 × 16 real matrix in this basis. Then the eigenvalues will still be either all real or some of them form complex conjugate pairs. However, unlike the single-qubit case, the number of complex conjugate pairs can be more than 1. Then, we may classify the quantum channel into different phases by the number of conjugate pairs, and the exceptional point will only occur at a phase transition. This implies exceptional points in higher-dimensional quantum channels can be more sophisticated and richer than the single qubit case and are worth investigating in further studies. With the EP order and therefore the sensitivity of the EP sensor potentially scalable with the number of interpolated channels. The importance of understanding higher-dimensional quantum channels is also highlighted in recent studies of quantum thermal machines^31^, where two-qubit systems serve as models for quantum heat engines. In such systems, the interplay between exceptional points and the system dynamics could lead to novel ways of controlling the quantum thermal machines.

In conclusion, we have demonstrated a systematic framework for generating and studying exceptional points in quantum channels through linearly interpolating channels in ${\mathcal{K}}$-exact and ${\mathcal{K}}$-broken phases. Our experimental implementations on an NMR quantum computer achieved process fidelities all above 93%, confirming the existence of second-order exceptional points at predicted parameter values. By extending the interpolation to three quantum channels, we revealed the possible generation of exceptional lines and higher-order exceptional points, including a third-order EP at specific interpolation parameters. This scalable approach promises applications from enhanced quantum sensing, where exceptional points enhance measurement sensitivity, to novel control mechanisms in quantum technologies. This approach establishes quantum channel interpolation as a versatile tool for generating and studying exceptional points in open quantum systems. Beyond demonstrating new sources of exceptional points, our work provides a general framework for understanding phase transitions in quantum channels. Future work could explore these channel-based exceptional points in higher-dimensional systems.

## Methods

### Quantum channels decomposition

For the sake of completeness, we show how to decompose an arbitrary single-qubit quantum channel into two simpler quantum channels that only need one ancilla qubit to implement in a quantum computer. Those simpler quantum channels are called quasiextreme quantum channels, which belong to the closure of the set of extreme points of the set of single-qubit channels as defined in ref. ^50^. We will also follow this work closely to decompose the quantum channels. To proceed, we first introduce the Choi operator ${\mathcal{C}}$ of a quantum channel ${\mathcal{E}}$, which is defined as

14 $${\mathcal{C}}\left({\mathcal{E}}\right)=\left({\mathcal{E}}{\mathscr{\otimes }}{\rm{I}}\right)\left(\left|{\Phi }^{+}\right.\right\rangle \left\langle \left.{\Phi }^{+}\right|\right)$$

where $|{\varPhi }^{+}\rangle =(|00\rangle +|11\rangle )$ is the un-normalized maximally entangled state. The Choi operator ${\mathcal{C}}$ is a positive semi-definite operator if and only if ${\mathcal{E}}$ is a completely positive map. To proceed, we also need to introduce the adjoint $\hat{{\mathcal{E}}}$ of a quantum channel ${\mathcal{E}}$, which is defined by ${Tr}\left[{\mathcal{E}}{\left(A\right)}^{\dagger }B\right]={Tr}\left[{A}^{\dagger }\hat{{\mathcal{E}}}\left(B\right)\right]$. This definition implies a more explicit way to write the adjoint quantum channel $\hat{{\mathcal{E}}}$ in Choi operator form as

15 $${\mathcal{C}}\left(\hat{{\mathcal{E}}}\right)={\left({U}_{23}^{\dagger }\mathcal{C} \left({\mathcal{E}}\right){U}_{23}\right)}^{*}$$

where *U*_23_ is a permutation matrix swapping the 2nd and 3rd coordinates, i.e.,

16 $${U}_{23}=\left(\begin{array}{cccc}1 & 0 & 0 & 0\\ 0 & 0 & 1 & 0\\ 0 & 1 & 0 & 0\\ 0 & 0 & 0 & 1\end{array}\right)$$

With the above definition, we can start to decompose the quantum channel ${\mathcal{E}}$ into two quasiextreme quantum channels. First, we consider the Choi operator of the adjoint quantum channel $M={\mathcal{C}}\left(\hat{{\mathcal{E}}}\right)$. It is shown in ref. ^50^, for a general quantum channel, this Choi operator (in |00〉,|10〉,|01〉,|11〉 ordering) can be written as

17 $$M=\left(\begin{array}{cc}A & \sqrt{A}R\sqrt{I-A}\\ \sqrt{I-A}{R}^{\dagger }\sqrt{A} & I-A\end{array}\,\right)$$

for some 2 × 2 positive semi-definite matrix *A* and a contraction matrix *R*, i.e., with both singular values less than 1. For a quasiextreme channel, all singular values are 1, and hence *R* is now a unitary matrix U. We can decompose the contraction matrix *R* into two unitary matrices *U*_1_ and *U*_2_, for example, we can use singular values decomposition

$$R =V\left(\begin{array}{cc}\cos {\theta }_{1} & 0\\ 0 & \cos {\theta }_{2}\end{array}\,\right){W}^{\dagger }=\frac{1}{2}V\left(\begin{array}{cc}{e}^{i{\theta }_{1}} & 0\\ 0 & {e}^{i{\theta }_{2}}\end{array}\,\right){W}^{\dagger }+\frac{1}{2}V\left(\begin{array}{cc}{e}^{-i{\theta }_{1}} & 0\\ 0 & {e}^{-i{\theta }_{2}}\end{array}\,\right){W}^{\dagger }=\frac{1}{2}{U}_{1}+\frac{1}{2}{U}_{2}$$

With the above definition for *U*_1_, *U*_2_ we can start to decompose the quantum channel ${\mathcal{E}}$ into $\frac{1}{2}\left({M}_{1}+{M}_{2}\right)$ using two Unitary matrices *U*_1_ and *U*_2_ and Eq. (17) as above. We then further obtain two Choi matrices using *M*_1_ and *M*_2_ in Eq. (15) and obtain two quantum channels ${{\mathcal{Q}}}_{1}$ and ${{\mathcal{Q}}}_{2}$ which are quasiextreme quantum channels. Finally, we will check the validity of the decomposition by comparing the Choi operator of the original quantum channel ${\mathcal{E}}$ with the mean of the Choi operator of the two quasiextreme quantum channels ${{\mathcal{Q}}}_{1}$ and ${{\mathcal{Q}}}_{2}$.

With these two quasiextreme quantum channels, we can implement each of them using only a 2-qubit circuit, as shown in ref. ^45^. Here, we discuss the details of this implementation. For each quasiextreme quantum channel ${\mathcal{Q}}$ obtained above, we first consider the 4 × 4 matrix in Eq. (2). Here, we denote it as ${\mathcal{P}}({\mathcal{Q}})$ to differentiate it from its Choi operator ${\mathcal{C}}\left({\mathcal{Q}}\right)$. Now we have a distortion matrix *E* and a vector *s*. We then do singular value decomposition on *E* as $E={O}_{1}\Sigma {O}_{2}^{T}$ where *O*_1_ and *O*_2_ are orthogonal matrices, and Σ is a diagonal matrix with singular values. To cast the orthogonal matrices into the special orthogonal group SO(3), we redefine them as ${O}_{i}^{{\prime} }=\det \left({O}_{i}\right){O}_{i}$ for *i* = 1,2. We then decompose ${\mathcal{P}}({\mathcal{Q}})$ using the above decomposition as

18 $$\begin{array}{ll}{\mathcal{P}}\left({{\mathcal{Q}}}_{i}\right) \!\!&\!\! =\left(\begin{array}{cl}1 & 0\\ s & E\end{array}\,\right)\\ \!\!&\!\! =\left(\begin{array}{cl}1 & 0\\ 0 & {O}_{1}^{{\prime} }\end{array}\,\right)\left(\begin{array}{cc}1 & 0\\ {s}^{{\prime} } & {E}^{{\prime} }\end{array}\,\right)\left(\begin{array}{cc}1 & 0\\ 0 & {{O}_{2}^{{\prime} }}^{T}\end{array}\,\right).\end{array}\,\,$$

Where ${s}^{{\prime} }={O}_{1}^{{\prime} T}s$ and the ${E}^{{\prime} }={{O}_{1}^{{\prime} }}^{{\rm{T}}}E {O}_{2}^{{\prime} }$. For the quasiextreme quantum channel, the diagonalized distortion matrix *E*' and modified shift vector *s*' are parameterized by

19 $${E}^{{\prime} }={\rm{diag}}\left(\cos \nu ,\cos \mu ,\cos \nu \cos \mu \right),$$

20 $${s}^{{\prime} }={\left(0,0,\sin \nu \sin \mu \right)}^{T}$$

for some *μ* and *ν*. With this factorization, we can implement the quasiextreme quantum channel by implementing each of the factorized matrix in Eq. (18) For the first and last matrix, the pure rotation then can be implemented by a single qubit gate *U*(*δ*) and *U*(*ϕ*) as where *δ* and *ϕ* are the Euler angles of the rotation matrix ${O}_{1}^{{\prime} }$ and ${O}_{2}^{{\prime} T}$ respectively. The middle matrix can be implemented by 2 CNOT gates with ${R}_{y}\left(2\gamma \right)$ and ${R}_{y}\left(2\sigma \right)$ gates as shown in Fig. 2a, where $2\gamma =-\nu +\pi /2$ and $2\sigma =\mu -\pi /2$.

### Fitting procedure and calculation of quantum channel fidelity

In this appendix, we describe the fitting procedure for QPT, which aims to reconstruct the quantum channel ${\mathcal{E}}$ that best describes an applied quantum operation. We assume the ability to prepare six perfect input states $|{\psi }_{i}\rangle \in \{|{x}_{\pm }\rangle ,|{y}_{\pm }\rangle ,|{z}_{\pm }\rangle \}$, represented in the computational basis as:$|{x}_{\pm }\rangle =(|0\rangle \pm |1\rangle )/\sqrt{2}$, $|{y}_{\pm }\rangle =(|0\rangle \pm i|1\rangle )/\sqrt{2}$, $|{z}_{+}\rangle =|0\rangle$, $|{z}_{-}\rangle =|1\rangle$. For each input state, we apply the quantum operation to obtain the corresponding output state ${{\rho }_{i}}^{{\prime} }=E(|{\psi }_{i}\rangle \langle {\psi }_{i}|)$. We then perform projective measurements on each output state, projecting onto the same set of six states $|{\phi }_{i}\rangle \in \{|{x}_{\pm }\rangle ,|{y}_{\pm }\rangle ,|{z}_{\pm }\rangle \}$. This results in 36 experimental measurement outcomes ${M}_{{ij}}^{(\exp )}=\langle {\phi }_{j}|{{\rho }_{i}}^{{\prime} }|{\phi }_{j}\rangle$.

To reconstruct the quantum channel, we work in the Choi matrix representation. The Choi matrix ${{\mathcal{C}}}_{{\mathcal{E}}}$ to be determined has the form:

$${{\mathcal{C}}}_{{\mathcal{E}}}=\left(\begin{array}{cccc}{{\rm{C}}}_{11} & {{\rm{C}}}_{12} & {{\rm{C}}}_{13} & {{\rm{C}}}_{14}\\ {{\rm{C}}}_{21} & {{\rm{C}}}_{22} & {{\rm{C}}}_{23} & {{\rm{C}}}_{24}\\ {{\rm{C}}}_{31} & {{\rm{C}}}_{32} & {{\rm{C}}}_{33} & {{\rm{C}}}_{34}\\ {{\rm{C}}}_{41} & {{\rm{C}}}_{42} & {{\rm{C}}}_{43} & {{\rm{C}}}_{44}\end{array}\right)$$

With this representation, we can calculate the expected measurement outcome ${M}_{{ij}}({{\mathcal{C}}}_{{\mathcal{E}}})$ by first converting the Choi matrix to its superoperator representation

$${{\mathcal{S}}}_{{\mathcal{E}}}=\left(\begin{array}{cccc}{{\rm{C}}}_{11} & {{\rm{C}}}_{31} & {{\rm{C}}}_{13} & {{\rm{C}}}_{33}\\ {{\rm{C}}}_{21} & {{\rm{C}}}_{41} & {{\rm{C}}}_{23} & {{\rm{C}}}_{43}\\ {{\rm{C}}}_{12} & {{\rm{C}}}_{32} & {{\rm{C}}}_{14} & {{\rm{C}}}_{34}\\ {{\rm{C}}}_{22} & {{\rm{C}}}_{42} & {{\rm{C}}}_{24} & {{\rm{C}}}_{44}\end{array}\right),$$

then computing:

$${M}_{{ij}}\left({{\mathcal{C}}}_{{\mathcal{E}}}\right)=\mathrm{vec}{\left(\left|{\phi }_{j}\right\rangle \left\langle {\phi }_{j}\right|\right)}^{\dag}{{\mathcal{S}}}_{{\mathcal{E}}}\mathrm{vec}\left(\left|{\psi }_{i}\right\rangle \left\langle {\psi }_{i}\right|\right)$$

where vec is the vectorization operation and $\mathrm{vec}\left(|{{\rm{\psi }}}_{i}\rangle \langle {{\rm{\psi }}}_{i}|\right)$ and $\mathrm{vec}\left(|{{\rm{\phi }}}_{j}\rangle \langle {\phi }_{j}|\right)$ are known constants. Since this expression is linear in the elements of ${{\mathcal{C}}}_{{\mathcal{E}}}$, we can perform a least-squares fit by minimizing ${{||}{M}_{{ij}}\left({{\mathcal{C}}}_{{\mathcal{E}}}\right)-{M}_{{ij}}^{\left(\exp \right)}{||}}^{2}$, where ${M}_{{ij}}\left({{\mathcal{C}}}_{{\mathcal{E}}}\right)$ and ${M}_{{ij}}^{\left(\exp \right)}$ are treated as vectors.

To ensure the reconstructed channel is physically valid, we impose several constraints. First, to guarantee complete positivity, ${{\mathcal{C}}}_{{\mathcal{E}}}$ must be Hermitian, positive semi-definite (${{\mathcal{C}}}_{{\mathcal{E}}}={{\mathcal{C}}}_{{\mathcal{E}}}^{\dagger },\,{{\mathcal{C}}}_{{\mathcal{E}}}\ge 0$). Also, to ensure trace preservation, we require ${C}_{11}+{C}_{22}={C}_{33}+{C}_{44}=1$ and ${C}_{13}+{C}_{24}={C}_{31}+{C}_{42}=0$. Combining these requirements yields the following optimization problem:

$$\begin{array}{ll}{\mathrm{minimize}} & {\left|\left|{M}_{ij}\left({{\mathcal{C}}}_{{\mathcal{E}}}\right)-{M}_{ij}^{\left(\exp \right)}\right|\right|}^{2}\\ {\mathrm{subject}}\,{\mathrm{to}} & {{\mathcal{C}}}_{{\mathcal{E}}}={{\mathcal{C}}}_{{\mathcal{E}}}^{\dagger },\\ & {{\mathcal{C}}}_{{\mathcal{E}}}\ge 0,\\ & {C}_{11}+{C}_{22}={C}_{33}+{C}_{44}=1,\\ & {C}_{13}+{C}_{24}={C}_{31}+{C}_{42}=0.\end{array}$$

We solve this optimization using CVXPY, a Python-based convex optimization solver.

Finally, to assess the quality of the reconstructed quantum channel, we calculate the channel fidelity between the target Choi matrix ${{\mathcal{C}}}_{\mathrm{tar}}$ and the reconstructed Choi matrix ${\mathcal{C}}$ using

$${Tr}{\left[\sqrt{{{\mathcal{C}}}_{\mathrm{tar}}}{\mathcal{C}}\sqrt{{{\mathcal{C}}}_{\mathrm{tar}}}\right]}^{2},$$

which is reported in Fig. 3.

## Data Availability

The datasets generated and/or analyzed during the current study are available in the Zenodo repository, 10.5281/zenodo.18485169.

## Code availability

The code for quantum channels decomposition and quantum channel tomography used in this study is available in the Zenodo repository, 10.5281/zenodo.18485169.
